# An Exposition of the Pathology of Hysteria

**Published:** 1842-04

**Authors:** 


ART. VII.—1. *An Exposition of the Pathology of Hysteria: elucidated by a reference to the Origin, &c. of Hysterical Amaurosis.* By EDWARD OCTAVIUS HOCKEN, M.D.—*London*, 1842. 8vo, pp. 32.

2. *An Essay on the Influence of Constitution in the Production of Disease.* By EDWARD OCTAVIUS HOCKEN, M.D.—*London,* 1841. 8vo, pp. 64.

OUR principal reason for noticing these works of Dr. Hocken is to afford ourselves an opportunity of advising the author—who appears to have talent, and is doubtless young—to be more chary, for the future, of printing his lucubrations, unless they contain a considerably greater share of important or novel matter than the present ones can lay claim to. In the "Pathology of Hysteria," we do not find a single observation with which every physician is not already familiar, and which we have not met with, times out of number, in other publications. What conceivable inducement can any one have to consult the small and trivial pamphlet of Dr. Hocken, when he can find the same subject more methodically, thoroughly, amply, and efficiently treated of elsewhere? Does Dr. Hocken mean to call the cases given at pp. 14 and 16 hysteric? The former seems most probably to have been of the nature assigned by Mr. Tyrrell; and the latter was most distinctly *not* of the nature which Dr. Hocken would imply.

The "Essay on the Influence of Constitution in the Production of Disease" is still more unsatisfactory than its twin brother, being little else than vague and inaccurate declamation—inaccurate both as regards style and the statement of clinical facts. Of the truth of this remark a single quotation will, we are persuaded, satisfy the reader:

"Soon, however, the disease (Hydrophobia) developes itself, and, then, its characteristic sign is fully formed, he cannot endure the sight or sound of liquids, they produce horrible convulsions, the muscles of deglutition are hurried into spasmodic action, and the feeling of suffocation is intolerable. And not only do liquids thus agitate the death-claimed patient, but even the bare resemblance of sound or look, a looking-glass, the motion of earthenware, or even the zephyr's gentle play, call forth the anger of the nervous system of the patient, and then in a few short days kill him outright by their intensity, either during one of those fierce struggles of the nervous system for mastery, or wear him out by pure exhaustion, sinking, and collapse." (pp. 63-4.)

Dr. Hocken must be aware that many deaths from hydrophobia are recorded, in which, during the whole course of the disease, the symptom which gives its name to the malady (water-fear) was never manifested.

